# 

**Published:** 1891-06

**Authors:** 


### LITERARY.

MATERIA MEDICA AND THERAPEUTICS.—Being Vol. II., and comprising an independent one upon Drugs; by John V. Shoemaker, A. M., M.D., Professor of Materia Medica, etc., etc.

This present volume is a valuable addition to a complete medical library, not only useful but absolutely necessary to the enlightened practitioner, who aims to familiarize himself with the properties and value of drugs employed in ministering to the sick and the afflicted.

It is indeed, a complete and comprehensive statement of the elements, preparation, value and use of all the known drugs employed as curative agents in the various diseases that flesh is heir to, and the usual and proper doses for infants and adults, in their administration. The book is so thoroughly indexed that no loss of time occurs in getting at the information desired. No physician should be without it, 1000 pp. Royal octavo. F. A. Davis, Publisher, Philadelphia. Cloth $3.50, Sheep $4.50 (net). Branch Offices : New York, Chicago, Atlanta and London.

WOMAN AND HEALTH.—A Mother's Hygienic Hand Book, etc., etc. By M. Augusta Fairchild, M.D., 380 pp. octavo.

Who so completely as a woman, can comprehend the needs of women in health, in sickness and in suffering ? The above book is a revelation, and touches woman at every point, from the cradle to the grave, throughout all the changes and vicissitudes of life, whether regular and normal, or irregular and abnormal. Much needed information is conveyed by way of dialogue, which enables the author to give a delicate shading to subjects usually but half explained. As a guide to health and mother's assistant, the work is unique. Published by the author, and on sale at the Fowler & Wells Co. ; price $2.50.

FEVER ; ITS PATHOLOGY AND TREATMENT.—By Prof. Hobart A. Hare, M. D., B. Sc. F. A. Davis, Medical Publisher, Philadelphia, 166 pp., price $1.25 net.

This is No. 10, of the Physician's and Students' Ready Reference series, illustrated with tables and charts, showing the state of the patient and the effect of the treatment, in the different stages of various fever cases, also the effect upon certain animals, of doses of antifebrin, thus literally carrying out the advice of the great Shakespere, to "Throw PHYSIC to the DOGS," in many instances, a most wise injunction, but particularly hard upon the dogs. It is not likely, however, that the immortal bard meant to include *good dogs!* The book will be found of great service to the actively employed physician.

MEDICAL SYMBOLISM, by Thos. Sozinskey.—Is a work of great interest, not only to the medical student, but to literary and scientific readers generally. The study of ancient symbolism is both interesting and instructive. The author gives sketches of the early worship of the serpent and its signification in the healing art, from the days of Æsculapius the "Father of Medicine," besides much valuable information, pertaining to medicine and hygiene of very ancient date. By the untimely death of the author, the literary world has lost one of its most useful members.—F. A. Davis, Phila., Publisher ; 171 pp., illust., price $1.00 net.

We have been favored with the following Essays, by their several authors.

HOW SHOULD GIRLS BE EDUCATED ? By Wm. Warren Potter, M.D., of Buffalo. The Anniversary address, N. Y. Medical Society, 1891.

FREE TRADE IN BANKING, and also THE FINANCIAL PROBLEM. By Alfred E. Westrup, Chicago, 1891.

LONG ISLAND COLLEGE HOSPITAL, BROOKLYN ; 33rd Annual Announcement. From which it appears that the Medical Class of the present year, numbered 250 ; the graduating class 82. 20,830 patients were under treatment in the hospital and dispensary, during the year 1890. The regular course of lectures will hereafter be six months in duration, and three courses will be required for graduation.

THE PUBLIC HEALTH, with special reference to Leprosy and Vaccination. By William Tebb, London, Eng.

OUR LITTLE MEN AND WOMEN.—Is always a most welcome visitor. The publishers, D. Lothrop Co., Boston, publishers also of BABY LAND, offer to send a specimen (back number) for 5 cents. $1.00 a year ; 10 cts. a number.

